# Glycine encephalopathy

**DOI:** 10.1186/s41983-022-00567-6

**Published:** 2022-11-17

**Authors:** S. Bhumika, Kanthesh M. Basalingappa, T. S. Gopenath, Suman Basavaraju

**Affiliations:** 1 Division of Molecular Biology, School of Life Sciences, JSS Academy of Higher Education and Research, Mysuru, 570015 India; 2 Department of Biotechnology and Bioinformatics, School of Life Sciences, JSS AHER, Mysuru, 570015 India; 3 Department of Periodontology, JSS Dental College and Hospital, S.S. Nagar, Mysuru, 570015 India

**Keywords:** Non-ketotic hyperglycinaemia, SLC6A9 gene, GLYT1 encephalopathy, MLPA, 13C glycine breath method

## Abstract

Inherited neurotransmitter diseases are a subset of rare neurometabolic disorders characterized by hereditary deficiencies in neurotransmitter metabolism or transport. Non-ketotic hyperglycinaemia (NKH), called glycine encephalopathy, is an autosomal recessive glycine metabolism disorder characterized by an abnormal accumulation of glycine in all bodily tissues, including the CNS. The SLC6A9 gene, which codes for the GLYT1 protein, a biochemical abnormality in the GCS, and dihydrolipoamide dehydrogenase enzymes, which function as a GCS component, are responsible for the neonatal form’s symptoms, which include progressive encephalopathy, hypotonia, seizures, and occasionally mortality in the first few days of life. By changing the MAPK signalling pathways, glycine deprivation in the brain damages neurons by increasing NMDA receptor activation, increasing intracellular Ca levels, and leading to DNA breakage and cell death in the neuron region. In addition to the previously mentioned clinical diagnosis, NKH or GE would be determined by MLPA and 13C glycine breath tests. Pediatricians, surgeons, neurologists, and geneticists treat NKH and GE at the newborn period; there is no cure for either condition.

## Introduction

Endogenous chemical messengers known as neurotransmitters improve the synaptic connection between neurons that are primarily engaged in a variety of physiological and psychological processes. Rare neurometabolic conditions known as inherited neurotransmitter disorders (NTDs) include enzymes involved in the synthesis of neurotransmitters or their required co-factors [1]. In these NTDs, one of the rare NTDs is glycine encephalopathy, called non-ketotic hyperglycinaemia. It is an autosomal recessive disorder marked by impaired glycine cleavage enzyme activity [2]. Mabry and colleagues originally described non-ketotic hyperglycinaemia in two newborns from the same sibship in 1963, presenting with severe neurological discomfort, hypotonia, frequent convulsions, and obtundation.

The syndrome is unique from Childs and colleagues [3] previously documented hyperglycinaemia with acidosis (1961). Glycine concentrations in plasma are elevated in a range of metabolic diseases in infants and children. In the ketotic-hyperglycinaemia condition, elevated blood levels of glycine seem to be involved in the accumulation of one or more unusual organic acids produced by the breakdown of amino acids chain. Propionic acidaemia [4] and methylmalonic acidaemia [5] are two conditions that are often linked to hyperglycinaemia; however, patients with isovaleric acidaemia [6] and beta-ketothiolase deficiency have also been reported to have it [7]. The majority of children with hyperglycinaemia, however, are classified as having non-ketotic hyperglycinaemia since there are often no detectable abnormalities in organic acid metabolism.

Many people with this diverse collection of illnesses suffer a life-threatening sickness in their infants, characterized by intractable seizures, stiffness, severe mental retardation, and early mortality. The non-ketotic hyperglycinaemia condition has been treated in a variety of ways, but none of them have worked [8].

According to Rees and colleagues in 2003, the spinal cord circuit is where the glycine receptor (GlyR) performs its most well-known function. They are often located in the spinal cord, help with synaptic transmission [9–11], and are essential for both motor control and pain perception [12–14]. They first occur during the early stages of spinal cord development, and the growth of their subunit composition is controlled [15]. It’s interesting to note that GlyR has been demonstrated to influence the differentiation of interneurons and synaptogenesis in the spinal cord [11].

The glycine cleavage system is made up of the glycine decarboxylase gene, which codes for the P-protein, the amino methyltransferase gene, which codes for the T-protein, and the glycine cleavage system H-gene, which codes for the H-protein [16]. While people with variation NKH have changes in the genes that make up lipoate and its cofactor. In addition, the disruption of GlyRs activity results in disease of the brain.

In individuals with side epilepsy, alternative splice variants have been identified [17]. Additionally, a mutation in the gene encoding the amino methyltransferase enzyme (AMT), also known as the Glyr-2 subunit [18], is present in autistic patients and is a significant contributor to glycine breakdown [19]. Five symmetrically organized subunits of the trans-membrane protein complexes known as GlyRs are positioned around a central pore. So far, five kinds of GlyR subunits have been identified: four alphas and one beta [8, 20]. For some alpha subunits, alternate splicing can result in novel variants [21–24]. Alpha subunits can produce homomeric or heteromeric receptors when they are coupled with the beta subunit [25, 26].

Glycine may be investigated as an amino acid that can aid COVID-19 patients reduce tissue damage and cytokine storm, according to Chuan Yuan Li., 2020 suggestion [27]. Glycine protects cells from pyroptosis and the production of proinflammatory cytokines by binding to its receptor GlyR, inducing a chloride influx that results in cellular membrane hyperpolarization.

If glycine is successful in clinical trials, it can be given to COVID-19 patients everywhere in the globe right now.

### Aetiology

Glycine builds up in the brain as a result of defects in the glycine cleavage pathway in NKH, an autosomal recessive disorder, as was previously mentioned [28]. Glycine accumulation in the brain changes the MAPK signalling pathways, which injures neurons [29]. Glycine activates N-methyl-d-aspartate receptors in the cerebral hemispheres and cerebellum, but it inhibits N-methyl-d-aspartate receptors in the brainstem and spinal cord [30, 31]. 5, 10-Methylenetetrahydrofolate, a byproduct of glycine hydrolysis, is also necessary for DNA synthesis. Since neural stem cell proliferation depends on the glycine cleavage pathway, it is highly expressed throughout development [32].

In NKH, a wide range of neurological dysfunctions, including severe infant hypotonia, failure to thrive, minor mental impairment, and learning challenges. The most severe and frequent form of NKH is called a classic form, which is characterized by infant encephalopathy, respiratory problems, and multifocal myoclonic convulsions. In those with classical NKH, GLDC gene mutations can occur in up to 75% of cases [33]. Less than 1% and 20% of instances of NKH, respectively, are caused by mutations in the GCSH and AMT genes [33, 34]. Medical symptoms are significantly influenced by the quantity of remaining glycine cleavage system hobby [28, 35]. The infantile shape typically includes seizures after 6 months of life.

Patients with late-onset NKH commonly show typical intellectual traits, along with spastic diplegia and optic atrophy [36]. The literature has also identified a transitory form of NKH, in which biochemical and electroencephalogram (EEG) abnormalities resolve by 2 months of age [37, 38]. In situations where the hepatic and cerebral glycine cleavage systems did not mature in a timely manner, full medical judgments are made. There have been reports of anatomical brain deformities, aberrant white matter signalling, and progressive brain atrophy. Patients that have NKH [39, 40]. The function of glycine in neural stem cells and synaptic transmission proliferation [41]. Callosal corpus, one of the characteristics of NCH-related deformities is dysgenesis. Involvement of the substantia Nigra, red nucleus, thalamus, deep grey matter nucleus, and ventral brainstem tract has also been reported.

### Pathophysiology

**Fig. 1:**
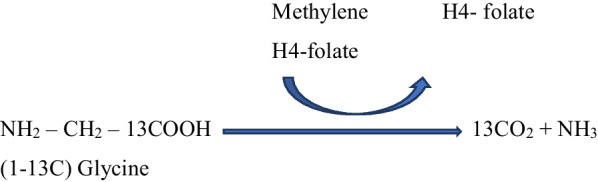
Enzymatic reaction of GCS. Glycine breaks down into carbon dioxide, ammonia, and one carbon atom

A significant increase in glycine concentration in cerebrospinal fluid was associated with NKH. It exhibits that glycine build-up in the brain is caused by a glycine cleavage device failure. A proportion of coronary artery disease (CAD) patients have been shown to have excessive amounts of glycine in their brains [42, 43]. The GCS is a mitochondrial multi-enzyme complex found in the brain, liver, kidney, and testis that catalyses the breakdown of glycine to carbon dioxide, ammonia, and one carbon unit [44], as shown in Fig. 1 [45].

Kure and colleagues in 2011 [46] say that GCS has four proteins: glycine dehydrogenase, amino methyltransferase, GCS H-protein, and dihydrolipoamide dehydrogenase which are encoded genes for each of these proteins, GLDC, AMT, GCSH, and DLD, respectively. The enzyme that is a part of other complex enzyme systems is called dihydrolipoamide dehydrogenase (DLD gene) (pyruvate dehydrogenases complex and the branched-chain ketoacid dehydrogenase complex).

Although NKH's neurological impairment is assumed to be caused by a high glycine level in the brain, its exact pathophysiology is yet unknown. However, several studies have helped us understand the relationship between glycine and neuroexcitotoxicity. Glycine is now believed to act as an inhibitory neurotransmitter at a strychnine-touchy receptor [47–51]. According to Johnson and Ascher and colleagues in 1987 [52], the glutaminergic receptor N-methyl-d-aspartate (NMDA) is potentiated by the excitatory property of glycine.

Based on experimental findings with cultured mouse brain neurons, it has been determined that glycine increases NMDA-mediated responses at a location roughly connected to the NMDA receptor. The NMDA receptor possesses excitatory amino acid receptors that are hyperactive, according to McDonald and Johnston and colleagues in 1992 [53], which have been linked to the pathophysiology of neuronal damage in a number of neurological conditions such hypoxia, ischaemia, hyperglycinaemia, or physical brain trauma. Growing neurophysiological evidence is in favour of this.

Sato and colleagues in 1991 [54] studied GCS studied rat brains utilizing P-protein antibody immunostaining, and discovered that the GCS was restricted to the astrocytes. The brain stem and spinal cord exhibited the least amount of astrocyte staining, whereas the hippocampus and cerebellar cortex had the most. In different parts of the brain, astrocyte staining varied. The fact that astrocytes in the CNS contain GCS indicates that glycine breakdown occurs mostly in these cells.

**Fig. 2:**
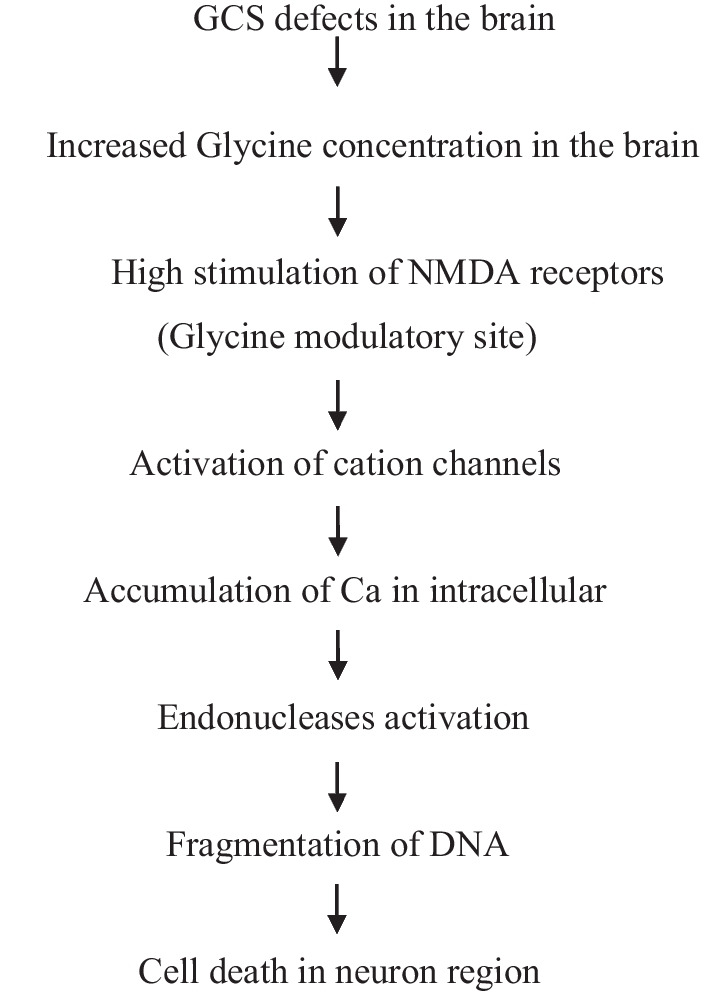
Pathophysiology of NKH

Because there is a link between the locations of the GCS and the NMDA receptors, it is seen that astrocytes of the GCS are associated with NMDA receptors. According to Tada K and colleagues in 1993 [55], the addition of glutamate causes the DNA chromosomal of the neurons to disintegrate into nucleosomal size DNA fragments, which results in glutamate-induced neuronal death (Fig. 2). Endonuclease inhibitors reduced DNA fragmentation and neuronal death, indicating that activating endonuclease is what causes DNA fragmentation. Instead of glutamate, this might cause an increase in intracellular Ca2 + concentration, which would then cause DNA fragmentation. The subcellular mechanism of neuroexcitotoxicity may thus be DNA fragmentation by glutamate, according to their studies.

### Genetics

According to Irene Mademont-Soler and colleagues in 2020 [56], a high concentration of glycine in the brain causes glycine encephalopathy, also known as glycine transporter 1 encephalopathy (GLYT1 encephalopathy). The protein GLYT1, which includes 14 exons, an intercellular N- and C-terminal, as well as 12 trans-membrane domains, is encoded by SLC6A9, according to Rayan Alfallaj and colleagues in 2019 [16]. When two Na and Cl ions bind to glycine, GLYT1 activity, or glycine transport at glial cells, is stimulated. In each of their case studies including GLYT1, Rayan Alfallaj and colleagues in 2019 [16] proposed an autosomal recessive inheritance pattern. A homozygous missense mutation in the SLC6A9 gene causes GLYT1, a deadly disease.

### Diagnosis

Glycine encephalopathy, according to Rayan Alfallaj and colleagues in 2019 [16], and Kure and colleagues in 2011 [46], can be diagnosed in neonates or babies who have seizures, muscle hypotonia, and lethargy that are not easily explained by an infection, trauma, hypoxia, or other frequent paediatric issues. Based on the clinical symptoms, high levels of glycine in the CSF (greater than 0.09), normal plasma, enzymes, and genetic tests for SLC6A9 mutations. Less than 0.04 is seen in both normal and ketotic-hyperglycinaemia. GE is characterized by an EEG finding of a burst suppression pattern during the first month of life.

The 13C glycine breath test and the multiplex ligation-dependent probe amplification (MLPA) are the two methods for the identification of significant deletions in DLD which were introduced by Kure and colleagues in 2011 [46] as a result of recent reports on improvements in GE diagnosis. Both approaches would make it easier for patients with hyperglycinaemia to confirm the diagnosis of GE.

### Treatment

Only supportive care is now provided as a treatment for the illness. The therapy of GLYT1 involves the involvement of paediatricians, neurologists, geneticists, genetic counsellors, dietitians, physiotherapists, occupational therapists, and orthopaedic surgeons. Children with glycine transporter 1 encephalopathy (GLYT1) are at risk for aspiration, malnutrition, and stunting because of issues with stomach incoordination and swallowing. A feeding tube, such as a gastrostomy tube, may be a successful therapy for many patients in order to guarantee that they consume enough calories and prevent recurrent aspirations. It's also a good idea to get your hearing and eyesight checked often. Last but not least, it is essential to consult orthopaedic surgeons, physiotherapists, and occupational therapists on a regular basis [46].

## Conclusion

NKH, also known as GE, inherited neurotransmitter diseases that are a subset of rare neurometabolic disorders characterized by hereditary deficiencies in neurotransmitter metabolism or transport on GCS. There is no cure for NKH or GE; it is treated in the neonatal stage by paediatricians, surgeons, neurologists, and geneticists. Glycine deficiency in the brain causes neuronal damage by altering MAPK signalling pathways, resulting in increased NMDA receptor activation and intracellular Ca buildup, as well as cell death in the neuron area due to DNA fragmentation. GE can be diagnosed using MLPA and 13C glycine breath testing, in addition to the previously mentioned clinical diagnosis.

## Data Availability

Not applicable.

## Abbreviations

NKH: Non-ketotic hyperglycinaemia
GE: Glycine encephalopathy
GCS: Glycine cleavage system
NTD: Neurotransmitter disorder
GlyR: Glycine receptor
GLYT1: Glycine transporter 1 encephalopathy
AMT: Amino methyl transferase enzyme
NMDA: N-Methyl-d-aspartate receptor
GCSH: Glycine cleavage system protein H
DLD: Dihydrolipoamide dehydrogenase
GLDC: Glycine dehydrogenase
MLPA: Multiplex ligation dependent probe amplification
CAD: Coronary artery disease
EEG: Electroencephalogram
CSF: Cerebrospinal fluid
